# Supplementary material for: Cardiac Applications of CRISPR/AAV-Mediated Precise Genome Editing
Source: bioRxiv. 2024 Dec 4:2024.12.03.626493. Preprint. [Version 1] doi: 10.1101/2024.12.03.626493 (PMC11642850; doi:10.1101/2024.12.03.626493)
Supplement: Supplement 1 [file media-1.pdf]

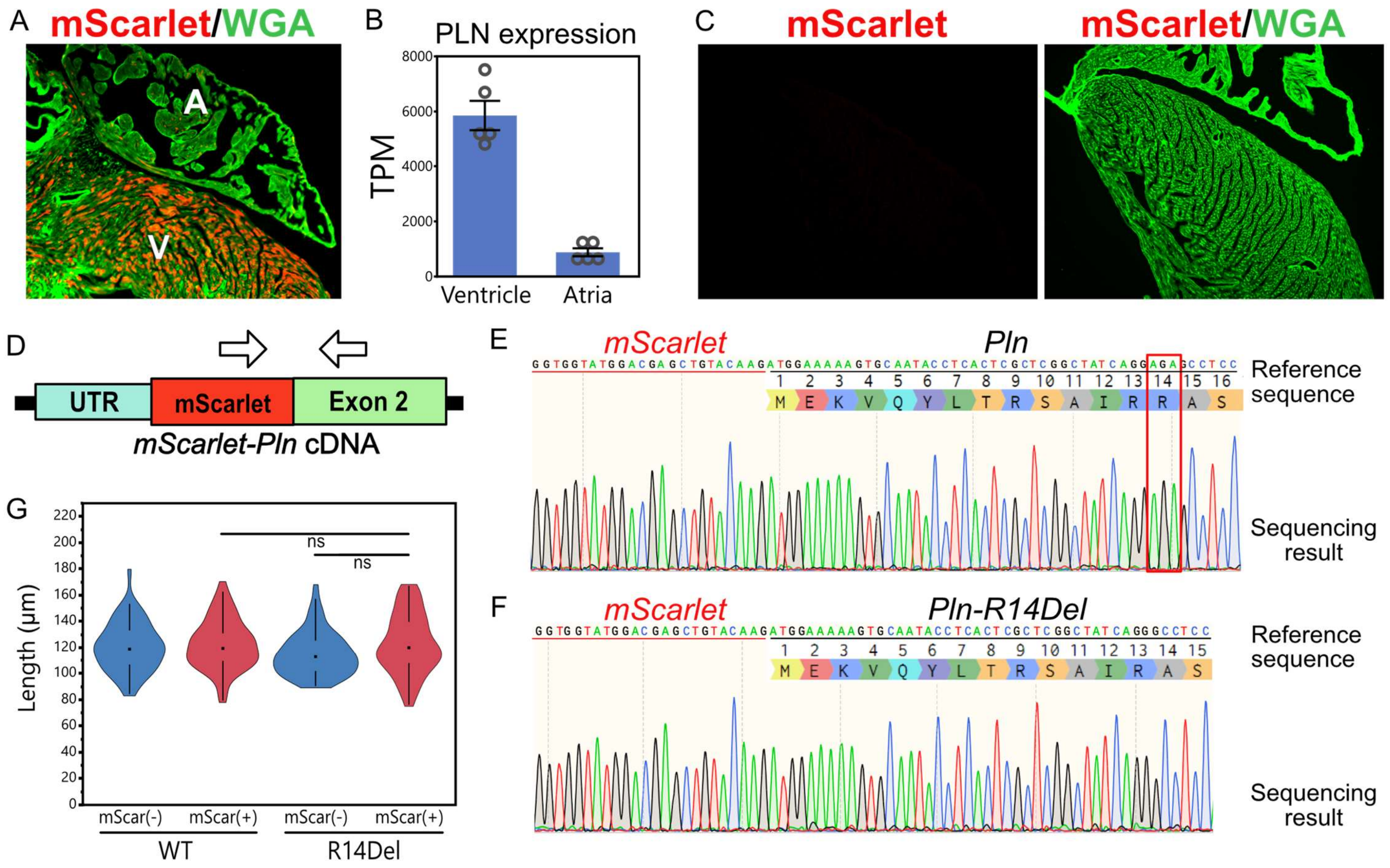

Suppl. Fig. 1. Generation and analysis of PLN R14Del cardiomyocytes A. Tissue section showing enriched mScarlet fluorescence in the ventricles (V) of PLN-WT hearts relative to the atria (A). **B.** Chamber specific *Pln* transcription in wildtype neonatal cardiomyocytes; mined from Cao et al. 2023. **C.** Cardiac tissue section from a wildtype (Cas9 negative) mouse injected with PLN-WT vector. **D.** Schematic of mScarlet/PLN junction to be Sanger sequenced from edited cDNA. **E.** Junction Sanger sequencing for PLN-WT hearts. Red box highlights R14. **F.** Junction Sanger sequencing for PLN-R14Del hearts. **G.** Length of isolated adult cardiomyocytes. Dunnett's *p* multiple comparison to R14Del mScarlet(+) group. ns, not significant. n = 56, 63, 63, and 62, for WT SN-, WT SN+, R14Del SN-, and R14Del SN+, respectively.

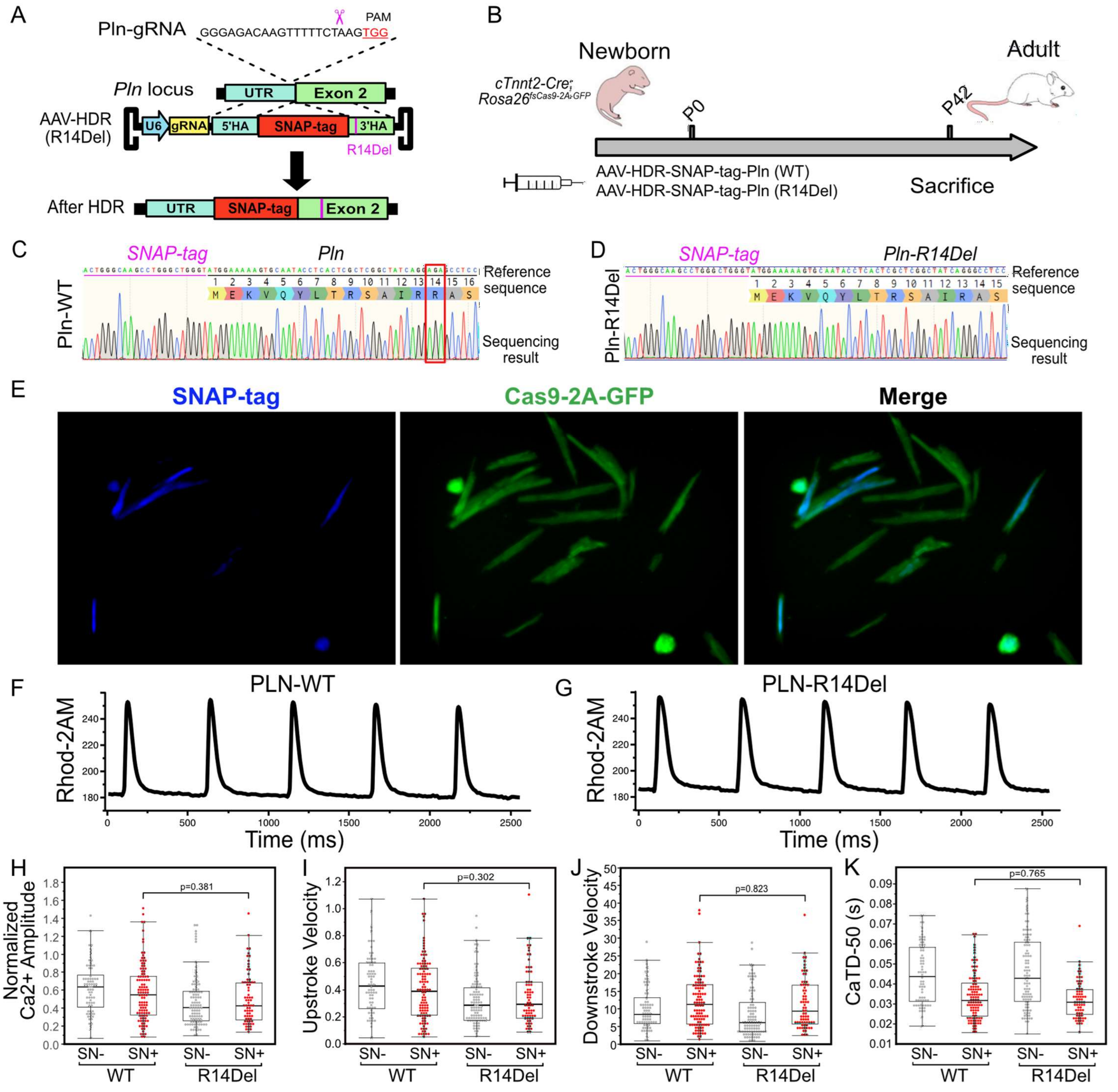

Suppl. Fig. 2. Calcium transient analysis in PLN R14Del cardiomyocytes. A. Homology directed repair-based strategy for fusing a SNAP-tag to wildtype and R14Del PLN. **B.** Experimental timeline. **C.** Sanger sequencing of cDNA from SNAP-Pln edited allele. **D.** Sanger sequencing of cDNA from SNAP-Pln-R14Del edited allele. **E.** SNAP-tag based far-red labeling of edited cardiomyocytes. Rhod-2AM labeled calcium transients were imaged in the red channel (not shown). **F.** Representative calcium trace for a PLN-WT cardiomyocyte. **G.** Representative calcium trace for a PLN-R14Del cardiomyocyte. **H.** Calcium transient peak amplitude. **I.** Calcium transient upstroke velocity. **J.** Calcium transient downstroke velocity. **K.** Action potential duration at 50% of the peak. Dunnett's *p* multiple comparison to R14Del mScarlet(+) group. For panels H-K, WT SN-, WT SN+, R14Del SN-, and R14Del SN+ n = 70, 106, 98, and 70, respectively.

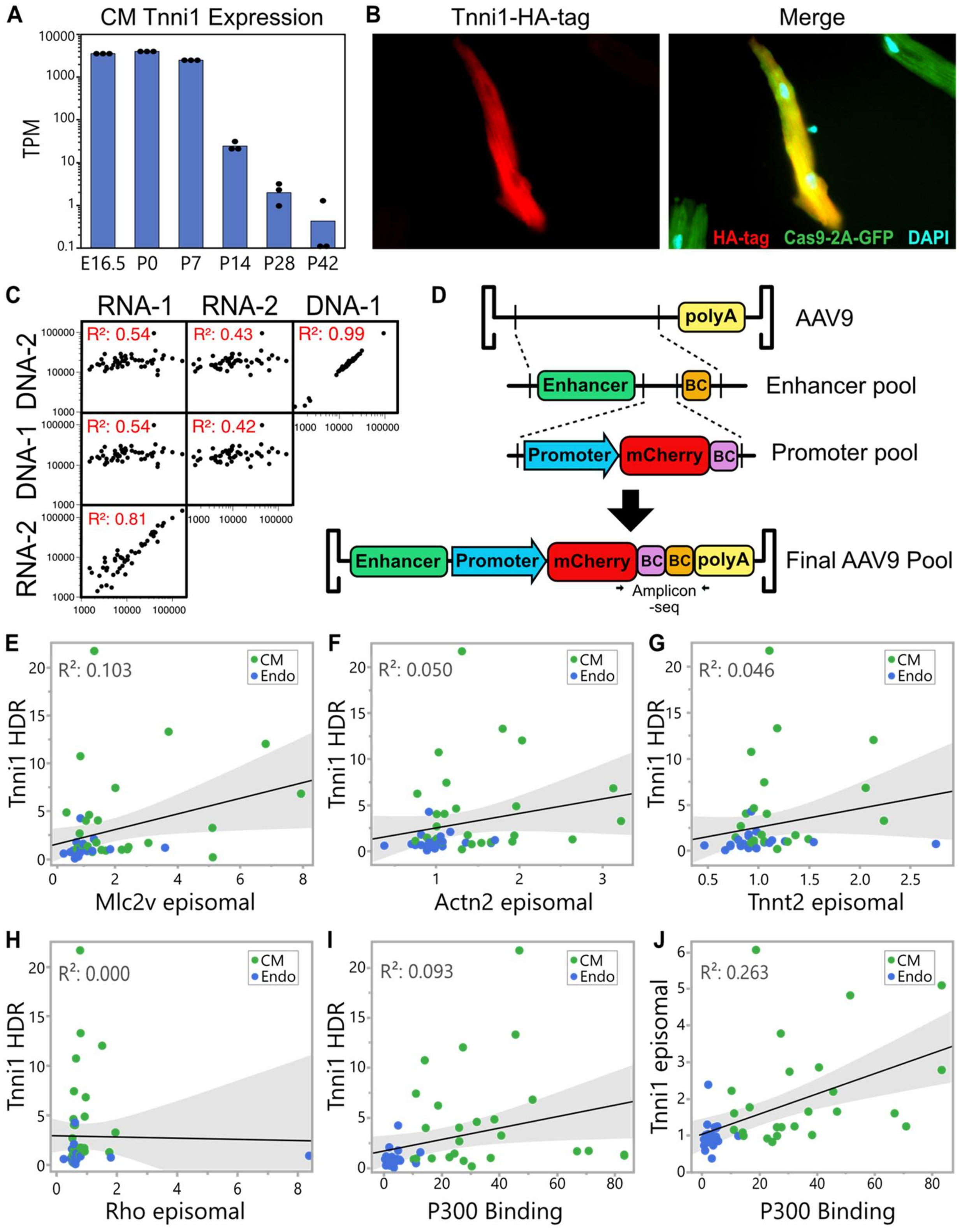

Supp. Fig. 3. Integrated and episomal MPRAs A. Tnni1 expression in cardiomyocytes. **B.** HA-tag stained cardiomyocytes from a mouse injected with Tnni1 enhancer-knockin vector. **C.** RNA and DNA samples were collected as duplicates. Sample-to-sample correlations of enhancer frequencies (FPM) are shown. **D.** Cloning strategy for episomal MPRAs. BC, barcode. **E-H.** Comparison of enhancer activities in Tnni1-HDR assay versus an episomal assay with various minimal promoters. **I, J.** Comparison of P300 occupancy at each native enhancer in adult cardiomyocytes to enhancer activities in the Tnni1 HDR assay or episomal assay respectively.

Supplemental table 1

Primers used for Sanger sequencing and amplicon-sequencing.

Primer	Sequence
TTN.+AT-F	CCACAGTCACTACTAAGTGCCAT
TTN.-T-F	GGACGATGGAGGTGCCAAAA
TTN.WT-F	AGTAGCCTGTGCCTTTACTGG
TTN-mScarlet-R	ACGCTTCCCAGCCCATTGTT
PLN-mScarlet-F	GTTGTACCCCGAGGACGG
PLN-Snap-F	CATCGAGGAGTTCCCTGTG
PLN-R	ATGCAGATCAGCAGCAGACAT
Episomal-MPRA-Round1-F	ACCGGCGGCATGGACGAGCT
Episomal-MPRA-Round1-R	CTGGAGTTCAGACGTGTGCTCTTCCGATCTAAGTCAGATGCTCAA GGGGC
Episomal-MPRA-Round2-F	AATGATACGGCGACCACCGAGATCTACACTCTTTCCCTACACGAC GCTCTTCCGATCTGCATGGACGAGCTGTACAAG
Episomal-MPRA-Round2-R (N = index)	CAAGCAGAAGACGGCATACGAGATNNNNNNGTGACTGGAGTTCA GACGT
Integrated-MPRA-RNA-Round1-F	ATCGATAATCGGCCGGCCGTC
Integrated-MPRA-RNA-Round1-R	CTGGAGTTCAGACGTGTGCTCTTCCGATCTTCCTCAGCCTAGGGC CCCTT
Integrated-MPRA-RNA-Round2-F	AATGATACGGCGACCACCGAGATCTACACTCTTTCCCTACACGAC GCTCTTCCGATCTTCGGCCGGCCGTCAGCGATC
Integrated-MPRA-RNA-Round2-R (N = index)	CAAGCAGAAGACGGCATACGAGATNNNNNNGTGACTGGAGTTCA GACGT
Integrated-MPRA-DNA-Round1-F	GTTCAAGCGTCCTCCCCTGC
Integrated-MPRA-DNA-Round1-R	CTGGAGTTCAGACGTGTGCTCTTCCGATCTTAGGGTCCCCCCAGT TTCCA
Integrated-MPRA-DNA-Round2-F	AATGATACGGCGACCACCGAGATCTACACTCTTTCCCTACACGAC GCTCTTCCGATCTTCGGCCGGCCGTCAGCGATC
Integrated-MPRA-DNA-Round2-R (N = index)	CAAGCAGAAGACGGCATACGAGATNNNNNNGTGACTGGAGTTCA GACGT

Supplemental table 2

Guide RNA sequences used in this study.

	Guide RNA target	Sequence
sgRNA sequences	TTN.+AT	AGAGAAAAGGGTCTGACCAG
	TTN.-T	GGAAGTTACAACTATCACCA
	TTN.WT	CGTTCTATGTAAGAGGGCCA
	PLN	GGGAGACAAGTTTTTCTAAG
	TNNI1	CTCTAAGCACCTCTACTGGG

Supplemental table 3

Antibodies and stains used in this study.

	**Antibody (species)**	**Product Number, Manufacturer**
Primary antibody	HA-Tag (Rb)	C29F4, Cell Signaling
	Caveolin 3 (Rb)	PA1066, Life Technologies
	Phospholamban (Rb)	14562s, Cell Signaling
	Phospho-Phospholamban (Rb)	8496s, Cell Signaling
	GAPDH (Ms)	MA5-15738, Thermo Fisher
Secondary antibody or dye	Gt-anti-Rb Alexa Fluor® Plus 555	A32732, Thermo Fisher
	Gt-anti-Rb Alexa Fluor® Plus 647	A32733, Thermo Fisher
	Gt-anti-Ms Alexa Fluor® Plus 647	A32728, Thermo Fisher
	Anti-Rabbit HRP	042-206, Protein Simple
	SNAP-Cell® 647-SiR	S9102S, NEB
	Hoechst	62249, Thermo Fisher
	DAPI	D1306, Thermo Fisher
	WGA Alexa Flour® 647	W32466, Thermo Fisher

Supplemental table 4. MPRA enhancers

Validated enhancers were selected for the MPRA from a search of the VISTA Enhancer Database and the literature (PubMed ID indicated). The cell type the enhancer was reported to be active in is indicated in column A. Enhancers were individually synthesized with a barcode, which was used for the episomal MPRA.

Enhancer Type	ID	Barcode	Reference coordinates (mm10)	Assayed coordinates (mm10)	Nearest Gene	Reference	Notes
Myocardium	1	CAGCTTTTGG	chr5:122092218-122094768	chr5:122092834-122093233	Myl2	Vista Enhancer Database #mm77	-
Myocardium	2	TGCAGGTTCG	chr18:75502363-75503667	chr18: 75502708-75503107	Ctif	Vista Enhancer Database #mm257	-
Myocardium	3	GTGGAGTACT	chr19:53715569-53717446	chr19:53716451-53716850	Rbm20	Vista Enhancer Database #mm64	-
Myocardium	4	TTTCCGCCGG	chr11:65208855-65210457	chr11:65209431-65209830	Myocd	Vista Enhancer Database #mm67	-
Myocardium	5	TGGCCGCTGA	chr9:64044784-64046949	chr9:64045149-64045548	1700055C04Rik	Vista Enhancer Database #mm69	-
Myocardium	6	CTACAGACTC	chr8:33903821-33907069	chr8:33905770-33906169	Rbpms	Vista Enhancer Database #mm70	-
Myocardium	7	GTGTACGTTC	chr8:95426508-95428314	chr8:95426749-95427148	Cfap20	Vista Enhancer Database #mm71	-
Myocardium	8	ACCAACTTGG	chr3:152303123-152305850	chr3:152304424-152304823	Fam73a	Vista Enhancer Database #mm72	-
Myocardium	9	TCAAGGTTTG	chr12:83072395-83074413	chr12:83073211-83073610	Rgs6	Vista Enhancer Database #mm78	-
Myocardium	10	ATCCGCACCA	chr17:73022163-73024151	chr17:73022583-73022982	Lclat1	Vista Enhancer Database #mm82	-
Myocardium	11	CTAGTTAGGA	chr11:65648906-65651643	chr11:65649932-65650331	Map2k4	Vista Enhancer Database #mm85	-
Myocardium	12	GATGGGTGAA	chr15:66824301-66827162	chr15:66826151-66826550	Tg	Vista Enhancer Database #mm104	-
Myocardium	13	AAAGATGGAC	chr12:69757409-69759262	chr12:69757719-69758118	Cdkl1	Vista Enhancer Database #mm122	-
Myocardium	14	GTTAGTAACG	chr13:46571320-46574349	chr13:46571988-46572387	Cap2	Vista Enhancer Database #mm132	-
Myocardium	15	CTGGGTAGTC	chr1:184041270-184042762	chr1:184041892-184042291	Dusp10	Vista Enhancer Database #mm134	-
Myocardium	16	GGGGAGATCT	chr3:104596935-104600543	chr3:104599431-104599830	Slc16a1	Vista Enhancer Database #mm143	-
Myocardium	17	TACTCTTAAA	chr4:120391603-120393946	chr4:120392787-120393186	Scmh1	Vista Enhancer Database #mm145	-
Myocardium	18	AGGGTTGTCA	chr11:77422469-77424441	chr11:77423506-77423905	Ssh2	Vista Enhancer Database #mm146	-
Myocardium	19	GGTTGAACGA	chr18:80330119-80332361	chr18:80331066-80331465	Kcng2	Vista Enhancer Database #mm152	-
Myocardium	20	TCAAGCATGA	chr5:21329154-21331111	chr5:21329618-21330017	Ccdc146	Vista Enhancer Database #mm186	-
Myocardium	21	CACACGTCAA	chr17:48436330-48438535	chr17:48436592-48436991	Apobec2	Vista Enhancer Database #mm243	-
Myocardium	22	AATCTTACTT	chr14:63367789-63369845	chr14:63368465-63368864	Blk	Vista Enhancer Database #mm245	-
Myocardium	23	CAAGACCCAC	chr12:85454400-85456289	chr12:85455103-85455502	Fos	Vista Enhancer Database #mm260	-
Myocardium	24	GCATAAAAAA	chr7:110162809-110166321	chr7:110164032-110164431	Wee1	Vista Enhancer Database #mm725	-
Myocardium	25	TAACATCGAG	chr4:95317529-95318734	chr4:95317822-95318221	Fggy	Vista Enhancer Database #mm308	-
Endocardium	26	TCGCAAAGGG	chr2:26571520-26572509	chr2:26571854-26572253	Egfl7_E1	PMID28121289	-
Endocardium	27	GCGAATGATC	chr1:133491869-133494057	chr1:133493441-133493840	Sox13	Vista Enhancer Database #mm573	-
Endocardium	28	CGCCAATGCA	chr12:79795794-79798372	chr12:79796258-79796657	Rad51b	Vista Enhancer Database #mm1393	-
Endocardium	29	TCACGTCCTA	chr2:124554786-124555549	chr2:124554995-124555394	Sema6d	PMID28121289	-
Endocardium	30	GCAGGTCTGG	chr2:26578160-26579122	chr2:26578438-26578837	Egfl7_E3	PMID28121289	-
Endocardium	31	TAGCCCCCGT	chr4:139597441-139601336	chr4:139599079-139599478	Iffo2	Vista Enhancer Database #mm1506	Failed synthesis - not included
Endocardium	32	TTTCCTCAAA	chr2:26474735-26475664	chr2:26475026-26475425	Notch1_E1	PMID28121289	-
Endocardium	33	GAGCTCCCAG	chrX:48005714-48006741	chrX:48006092-48006491	Apln	PMID28121289	-
Endocardium	34	GAACCCTTAA	chr6:84048677-84049910	chr6:84048857-84049256	Dysf	Vista Enhancer Database #hs2170	Liftover from hg38 chr2:71490876-71494083
Endocardium	35	TGACCAAATT	chr4:135253467-135256150	chr4:135254797-135255196	Clic4	Vista Enhancer Database #mm1648	-
Endocardium	36	TACTTGAAGT	chr9:37399047-37402046	chr9:37400570-37400969	Robo4	PMID17495228	-
Endocardium	37	ATGCGTTGAA	chr4:94745331-94746949	chr4:94746449-94746848	Tek	PMID9096345	-
Endocardium	38	AGCCGTGCCG	chr11:49632161-49633018	chr11:49632269-49632668	Flt4	PMID19070576	-
Endocardium	39	TACATTACCA	chr8:128386820-128387840	chr8:128387203-128387602	Nrp1	PMID19070576	-
Endocardium	40	ATCGTGTTAT	chr4:137919926-137920531	chr4:137920023-137920422	Ece1	PMID19070576	-
Endocardium	41	AACATCTACC	chr4:115071093-115076331	chr4:115075532-115075931	Tal1	PMID14966269	-
Endocardium	42	AGTGAATAGC	chr2:119327125-119327925	chr2:119327153-119327552	Dll4	PMID23830865	-
Endocardium	43	CGGACAGTCG	chr6:88202604-88203797	chr6:88202958-88203357	Gata2	PMID17395646	-
Endocardium	44	CAGGAGAATG	chr13:83571928-83572369	chr13:83571999-83572398	Mef2c	PMID15501228	-
Endocardium	45	AAGTTATGCT	chr17:34564510-34565332	chr17:34564815-34565214	Notch4	PMID15684396	-
Endocardium	46	TGGGTTCTAG	chr19:37435671-37435904	chr19:37435625-37436024	Hhex	PMID15649946	-
Endocardium	47	TGGCAACTCA	chr5:75961867-75962690	chr5:75962135-75962534	Kdr	PMID27079877	-
Endocardium	48	TCTTACAGCG	chr18:13847776-13849408	chr18:13848506-13848905	Zfp521	Vista Enhancer Database #hs1653	Liftover from hg38 chr18:25228374-25230020
Endocardium	49	GGGGCACGCG	chr4:57845561-57847767	chr4:57846984-57847383	Pakap	Vista Enhancer Database #mm261	-
Endocardium	50	GGAGTATAAA	chr19:37492022-37497349	chr19:37493919-37494318	1700122C19Rik	Vista Enhancer Database #hs1866	Liftover from hg38 chr10:92754239-92758228
ESC	E1	GTGGTATGTG	N/A	chr2:4512536-4512936	Prpf18	PMID28121289	-
ESC	E2	GTTCTTCTGC	N/A	chr13:6040082-6040482	Klf6	PMID28121289	-
ESC	E3	ATTCTCTGCA	N/A	chr11:5046317-5046717	Rasl10a	PMID28121289	-
ESC	E4	GCTATGTAGG	N/A	chr7:4902457-4902857	Zfp628	PMID28121289	-
ESC	E5	AGACGCTAGA	N/A	chr8:3421492-3421892	Arhgef18	PMID28121289	-

Supplemental Table 5: MPRA promoters

Short promoter sequences overlapping the TSS of the indicated genes were selected for this study. Additional short minimal promoters were selected from the literature.

Promoter Name	Species	Sequence (5' --> 3')	Barcode	Source
Tnnt2	Mouse	TCTGAGCAGCTGGAGGACCACATGAGCTTATATGGCGTGGGGTACTTGTTCTTTTAGCCCTGTGCCGGGCACCTGCCAAAATAGCAGCCAACACCCCCCATTGTGTTGTTCCCCCCCCCCCCCATCTCCTGCTGCACATTCCTCCCTCCGCGGGGCTTGGCTCACAAGGCCCCAGCCCACATGCCTGCTTAAAGCTCTCC	TACGGCCTAG	this study
Tnnl1	Mouse	GCAGCACATATCTGCCCTGCGAGGTGTGCAGGGTGGGAGGGGGTGGGAAGGAGGGGCAGCTGGAGGGGCAGCGGCTGTTCTATTTTTACTGGCCAGTTGCCGGAGGCCACGGTTTTCATAGCCTGCCCTCAGCTCTGCCCCCACACTCTGCAGTCTGTGGTGAGGCTCAGGCCAGCCTAGCTCCACGAGGACTAAACTAG	TCCTAAATAA	this study
Actn2	Mouse	GGAACCTGAGCGGGGCTCTCCAGCCAGCCAACCACACGTGTTGGAGACGGGGAGTGGGTGGGGCCGGCAGCACGTGACTCTCAGCGGGCTATTAATCCGCGCGGCGCTGCCTGCAGGCGTGCTGGTACTTCGCCGGAGACTCCGCGCCCAGGCGTGCCGCCCCAGGAACCGCGAGAAGGTCACCGCAGCCCGCCGCCTTT	TATTCACGAA	this study
Rho	Cow	GATTCAGCCGGGAGCTTAGGGAGGGGAGGTCACTTCATAAGGGCTTGGGGGGGGAGTTGGAGCCACGAGTCGTCCAGCCGGAGCCCCGTGTGGCTGAGCTCCGGCCTCAGAAGCATCCCCGGGATCCACCGGTCGCCA	ATACACTGCG	PMID: 26576614
Mlc2v	Rat	AGACAATGGCAGGACCCAGAGCACAGAGCATCGTTCCCAGGCCAGGCCCCAGCCACTGTCTCTTTAACCTTGAAGGCATTTTTGGGTCTCACGTGTCCACCCAGGCGGGTGTCGGACTTTGAACGGCTCTTACTTCAGAAGAACGGCATGGGGTGGGGGGGCTTAGGTGGCCTCTGCCTCACCTACAACTGCCAAAAGTGGTCATGGGGTTATTTTTAACCCCAGGGAAGAGGTATTTATTGTTCCACAGCAGGGGCCGGCCAGCAGGCTCCTTGAATT	TAAATACTGT	PMID: 2808370

Supplemental table 6 - Activity for all enhancer-promoter pairs

Activity was calculated as the ratio of frequency in RNA/DNA, and normalized such that the average activity of the endothelial enhancers for each promoter was 1.0. Column "Tnni1UTR" acitivies are from the integrated MPRA, and "Mlc2v" to "Rho" are from the episomal MPRA. "P300 occupancy" reflects cardiomyocyte specific P300 occupancy at each enhancer region at P28.

Enhancer ID	mm10 coordinates	Enhancer Type	Tnni1UTR	Mlc2v	Tnni1	Actn2	TnT	Rho	P300 occupancy
1	chr5:122092218-122094768	Myocardial	4.880811	0.44483	1.015748	1.96218	-	0.933711	38.2306
2	chr18:75502363-75503667	Myocardial	4.629611	1.169266	1.251969	1.238042	0.951902	0.560226	32.1357
3	chr19:53715569-53717446	Myocardial	1.281334	2.427499	2.787402	2.6396	1.49217	1.748585	83.2251
4	chr11:65208855-65210457	Myocardial	1.748101	1.398036	1.251969	1.378198	1.029083	0.797898	70.9049
5	chr9:64044784-64046949	Myocardial	0.897714	1.703062	2.220472	1.541713	0.926174	0.780922	10.2621
6	chr8:33903821-33907069	Myocardial	1.359469	1.118429	5.102362	1.004449	-	0.933711	83.2194
7	chr8:95426508-95428314	Myocardial	4.053203	1.448873	1.228346	1.097887	0.87472	0.577203	26.0068
8	chr3:152303123-152305850	Myocardial	6.839292	7.943385	4.818898	3.130145	2.058166	0.967664	51.4636
9	chr12:83072395-83074413	Myocardial	0.753416	1.270942	1.228346	1.424917	0.951902	0.594179	27.5943
10	chr17:73022163-73024151	Myocardial	1.706236	3.062969	1.606299	1.938821	1.363535	0.916734	66.8625
11	chr11:65648906-65651643	Myocardial	0.975908	1.563258	1.606299	1.074527	0.951902	0.526273	11.1579
12	chr15:66824301-66827162	Myocardial	7.441511	2.008088	1.15748	1.121246	1.05481	0.577203	11.0729
13	chr12:69757409-69759262	Myocardial	10.75035	0.889659	1.086614	1.027809	0.926174	0.645109	14.1118
14	chr13:46571320-46574349	Myocardial	13.30717	3.71115	2.19685	1.798665	1.183445	0.797898	45.5615
15	chr1:184041270-184042762	Myocardial	0.205103	5.121895	2.740157	1.30812	1.183445	0.560226	30.3951
16	chr3:104596935-104600543	Myocardial	21.72777	1.334489	1.653543	1.30812	1.106264	0.780922	46.8825
17	chr4:120391603-120393946	Myocardial	4.023926	1.067591	0.992126	1.004449	1.080537	0.611156	14.435
18	chr11:77422469-77424441	Myocardial	12.03856	6.812247	3.779528	2.032258	2.135347	1.510914	27.3448
19	chr18:80330119-80332361	Myocardial	3.274014	5.109185	2.858268	3.223582	2.238255	1.952304	40.6119
20	chr5:21329154-21331111	Myocardial	6.249733	-	6.070866	0.770857	-	-	18.7383
21	chr17:48436330-48438535	Myocardial	2.694906	0.86424	0.992126	1.004449	0.823266	0.526273	26.0692
22	chr14:63367789-63369845	Myocardial	1.11813	0.775274	0.92126	0.747497	0.951902	0.611156	22.6674
23	chr12:85454400-85456289	Myocardial	1.042001	2.38937	1.653543	1.915462	1.05481	0.780922	36.9493
24	chr7:110162809-110166321	Myocardial	1.477818	0.940497	0.826772	0.887653	0.771812	0.577203	24.4079
25	chr4:95317529-95318734	Myocardial	0.998724	2.211438	1.771654	1.658509	1.286353	0.763945	16.5384
26	chr2:26571520-26572509	Endothelial	0.848888	1.105719	1.228346	1.027809	0.926174	0.662086	4.39967
27	chr1:133491869-133494057	Endothelial	4.274455	0.889659	0.968504	0.911012	0.926174	0.611156	4.87592
28	chr12:79795794-79798372	Endothelial	0.954853	0.915078	-	-	1.543624	-	4.36565
29	chr2:124554786-124555549	Endothelial	0.517701	-	-	-	0.720358	0.509297	11.0785
30	chr2:26578160-26579122	Endothelial	0.079795	0.711727	1.015748	0.887653	0.668904	0.628133	3.42448
32	chr2:26474735-26475664	Endothelial	1.793321	0.762565	1.204724	0.817575	0.900447	0.560226	4.87025
33	chrX:48005714-48006741	Endothelial	0.519217	0.775274	1.015748	1.074527	0.926174	0.662086	1.27001
34	chr6:84048677-84049910	Endothelial	0.928653	-	-	1.354839	-	8.369442	0.34018
35	chr4:135253467-135256150	Endothelial	1.607659	0.978625	0.992126	1.051168	0.900447	0.509297	12.5923
36	chr9:37399047-37402046	Endothelial	0.301802	0.749856	0.897638	0.887653	0.900447	0.526273	0.680361
37	chr4:94745331-94746949	Endothelial	0.525943	0.724437	0.826772	0.911012	0.823266	0.594179	2.35858
38	chr11:49632161-49633018	Endothelial	0.652498	0.87695	0.944882	0.957731	0.848993	0.628133	1.7009
39	chr8:128386820-128387840	Endothelial	0.657133	0.86424	0.850394	0.817575	0.823266	0.526273	3.56623
40	chr4:137919926-137920531	Endothelial	1.043112	1.842865	0.590551	1.074527	1.260626	-	1.08858
41	chr4:115071093-115076331	Endothelial	1.148077	0.902369	0.92126	0.98109	1.131991	0.645109	2.67609
42	chr2:119327125-119327925	Endothelial	2.103845	1.321779	1.299213	1.167964	0.977629	0.577203	1.8143
43	chr6:88202604-88203797	Endothelial	0.75082	0.571924	2.385827	-	2.752796	1.799515	2.15448
44	chr13:83571928-83572369	Endothelial	0.869685	1.270942	1.251969	1.658509	1.106264	0.797898	5.31815
45	chr17:34564510-34565332	Endothelial	0.279387	0.826112	1.03937	0.98109	0.977629	0.577203	2.09778
46	chr19:37435671-37435904	Endothelial	0.658034	0.673599	0.732283	0.747497	0.720358	0.475344	3.88373
47	chr5:75961867-75962690	Endothelial	0.773939	0.622761	0.850394	0.700779	0.848993	0.509297	5.60164
48	chr18:13847776-13849408	Endothelial	0.915348	0.66089	0.874016	0.911012	1.05481	0.594179	2.47765
49	chr4:57845561-57847767	Endothelial	0.59404	0.355864	0.377953	0.373749	0.463087	0.237672	3.41881
50	chr19:37492022-37497349	Endothelial	1.201792	3.596765	0.732283	1.705228	0.797539	-	0.725718

Supplemental Table 7 - Plasmid sequences

Experiments were conducted in vivo, with constructs delivered via standard recombinant AAV2 genome, packaged in an AAV9 capsid. Plasmid sequences, spanning from viral ITR to ITR, are included here for all experiments.

AAV-HDR-TTN.WT-mScarlet-Fusion - inserts mScarlet at C-terminus of wildtype Titin.
CTGCGCGCTCGCTCGCTCACTGAGGCCGCCCGGGCAAAGCCCGGGCGTCGGGCGACCTTTGGTCGCCCGGCCTCAGTGAGCGAGCGAGCGCGCAGAGAGGGAGTGGCCAACTCCATCACTAGGGGTTCCTTGTAGTTAATGATTAACCCGCCATGCTACTTATCTACCAGGGTAATGGGCT
CGAGGAGGGCCTATTTCCCATGATTCCTTCATATTTGCATATACGATACAAGGCTGTTAGAGAGATAATTGGAATTAATTTGACTGTAAACACAAAGATATTAGTACAAAATACGTGACGTAGAAAGTAATAATTTCTTGGGTAGTTTGCAGTTTTAAAATTATGTTTTAAAATGGACTATCATAT
GCTTACCGTAACTTGAAAGTATTTCGATTTCTTGGCTTTATATATCTTGTGGAAAGGACGAAACACCGCGTTCTATGTAAGAGGGCCAGTTTTAGAGCTAGAAATAGCAAGTTAAAATAAGGCTAGTCCGTTATCAACTTGAAAAAGTGGCACCGAGTCGGTGCTTTTTTGGTACCATTGTTCCT
GCAGGCAGATGTCACCGAGTAACGAGATGTCTTTGATGTCCCCCGTGTATCTGCTATGCTCTACAGCTCTGGTGGAAGAACCACCGAGAGAGGTGGTATTGAAAACGAGCAGTGACGTGAGCTTGCACGGAAGCGTCTCGTCTCAGTCAGTCCAAATGTCTGCCTCTAAGCAGGAGGCATCC
TTCAGCAGCTTCAGCAGCAGCAGCGCTAGCAGCATGACTGAAATGAAGTTTGCGAGCATGTCTGCCCAAAGCATGTCTTCCATGCAAGAGTCCTTTGTAGAAATGAGTTCCAGCAGTTTTATGGGAAAATCTAGCATGACACAACTGGAAAGCTCAACCAGTAGAATGCTCAAAGCAGGCGG
AAGAGGTGAGCAAAAGAGTTCCTGGTAGAGGTAGATCAAGCCACCTAGCATGGCATCGTTCTAGAGCAGTGGTTCTCCGCCTTCCTAATGCTGCGACCCTTTAATACAGTTCCTCACGTTGTCGAGACTCTCAATCATGAAATTATTTTTGTTGCTACTTCCCAACTGTAATTTTGCTAACTTTAT
GAGTCGTAATGTAAATATGTTTGGAGGTACGCGTTGGCCACAGGGGTCACGGCCCACATGTTGAGAACCACTGTCTTAGTGCATTAAAAGTCAATAGAAAGCGAATAGGATTTCTTGTTTTCGGTGACCCTAAAACTTTTCTGTTGCTGTTTCATAAAGGAATTCCACCTAAAATTGAAGCTCTT
CCCTCTGATATCAGCATCGACGAAGGCAAAGTCCTAACAGTAGCCTGTGCCTTTACTGGGGAGCCTACTCCAGAAATAACATGGTCCTGTGGTGGAAGGAAAATCCAAAACCAAGAACAACAAGGAAGATTCCACATTGAAAACACAGATGACCTGACCACCCTGATCATCATGGACGTACA
GAAACAAGATGGCGGACTTTATACCCTAAGCTTAGGGAATGAATTTGGATCTGACTCTGCCACTGTGAATATTAATATCCGTTCTATGATGGTGAGCAAGGGCGAGGCAGTGATCAAGGAGTTCATGCGGTTCAAGGTGCACATGGAGGGCTCCATGAACGGCCACGAGTTCGAGATCGAG
GGCGAGGGCGAGGGaCGCCCCTACGAGGGCACCCAGACCGCCAAGCTGAAGGTGACCAAGGGTGGCCCCCTGCCCTTCTCCTGGGACATCCTGTCCCCTCAGTTCATGTACGGCTCCAGGGCCTTCACCAAGCACCCCGCCGACATCCCCGACTACTATAAGCAGTCCTTCCCCGAGGGCTT
CAAGTGGGAGCGCGTGATGAACTTCGAGGACGGCGGCGCCGTGACCGTGACCCAGGACACCTCCCTGGAGGACGGCACCCTGATCTACAAGGTGAAGCTCCGCGGCACCAACTTCCCTCCTGACGGCCCCGTAATGCAGAAGAAaACAATGGGCTGGGAAGCGTCCACCGAGCGGTTGTA
CCCCGAGGACGGCGTGCTGAAGGGCGACATTAAGATGGCCCTGCGCCTGAAGGACGGCGGaCGCTACCTGGCGGACTTCAAGACCACCTACAAGGCCAAGAAGCCCGTGCAGATGCCCGGCGCCTACAACGTCGACCGCAAGTTGGACATCACCTCCCACAACGAGGACTACACCGTGGT
GGAACAGTACGAACGCTCCGAGGGtCGCCACTCCACCGGtGGtATGGACGAGCTGTACAAGTAGGAGGGCCAGGGCCAAGAGCGGGGGCCCTTTACACTCGATGCTCATTCATAATCTCTCTCAAATGATTCACATGAACTAATCTTTCTGACCTGTAAATATTTTTAAGTAGTTTTATATCCAT
CTGAATGAGTCACAGTTCCAAAACTATGCATTTCAATCCTTTCATACTTGACCTGAGAATATTTGCTAATGACACACATGTACATACTGTATATAGCCGGGTTCACGGTTATAAAGTTTTGTACCATTTTATTTTATGACATTTTACACTGTAACTTTTGAAACTAACTGTTGGTGGGAGAAAGTTTC
TTATAGATTATAGAACGAATACCCTGCCAATACTTAATCAATCTTTGTGCCTCAACATACTGTTGATGTCTAAGTATGCCTCAGTGGGTTGAGAAATTCCCCACTGAAGATGTCCTATCCACCTAAAAAAAAAAGGATGCTTCTGTACATGTCCCTGACATGTGCACTGACACCTATTGAATCAGA
AATGTAAGGCATTGGTGATGTTTGCATCTACCCTCCTGTAAGCAACACTTTAACGTCTTACGTTTTCTCTGATGATGTCACACTGAAAATTATCATGACAAATATAACCAGAGCAAAGTGTAACGGCCATACACTTTGTTCGCCCACTTCACACTGTCTCTGGCATACAGAGTGCATGGATAGCTT
GGGAAATGTACCATCTCCTGGCCATCCCCTCGTCTAACCAAGCTATGCAAGTATTCCTATGCCAGAGCGGTGTCGACTCCTGGAGGTCCCAGGTGCAGCTCCTGCCTTGTGTGGTAGTTCTAAATTCAAATTTCACCGGGAACTGGGCAACTAAGCAATGAGCCACAGCAAAATAATAATAATA
ATAATTTAAAAAAATGTAACAAGTGAAGCTGTCGTTAAAACAACAATACTACCAACTGCCCATAAAAAAAAAAAATTACTAATTTGATGTAGTCCTTTTCATGCCAGTATATGTTCAATTGGTAGTTATAAATGTTGCGATCGCACATGGGCTAGCGGACTTTGTACACTGGATACTAGTCCCATTA
CCCTGGTAGATAAGTAGCATGGCGGGTTAATCATTAACTACAAGGAACCCCTAGTGATGGAGTTGGCCACTCCCTCTCTGCGCGCTCGCTCGCTCACTGAGGCCGGGCGACCAAAGGTCGCCCGACGCCCGGGCTTTGCCCGGGCGGCCTCAGTGAGCGAGCGAGCGCGCAG

AAV-HDR-TTN.AT63117fs-mScarlet - inserts mScarlet after truncation caused by AT63117 mutation.
CTGCGCGCTCGCTCGCTCACTGAGGCCGCCCGGGCAAAGCCCGGGCGTCGGGCGACCTTTGGTCGCCCGGCCTCAGTGAGCGAGCGAGCGCGCAGAGAGGGAGTGGCCAACTCCATCACTAGGGGTTCCTTGTAGTTAATGATTAACCCGCCATGCTACTTATCTACCAGGGTAATGGGCT
CGAGGAGGGCCTATTTCCCATGATTCCTTCATATTTGCATATACGATACAAGGCTGTTAGAGAGATAATTGGAATTAATTTGACTGTAAACACAAAGATATTAGTACAAAATACGTGACGTAGAAAGTAATAATTTCTTGGGTAGTTTGCAGTTTTAAAATTATGTTTTAAAATGGACTATCATAT
GCTTACCGTAACTTGAAAGTATTTCGATTTCTTGGCTTTATATATCTTGTGGAAAGGACGAAACACCGAGAGAAAAGGGTCTGACCAGGTTTTAGAGCTAGAAATAGCAAGTTAAAATAAGGCTAGTCCGTTATCAACTTGAAAAAGTGGCACCGAGTCGGTGCTTTTTTGGTACCATTGTTCC
TGCAGGCTCTTGTTCAGATCCCCCAGGACCACCTTCAAATGCACATGTCACTGATACCACCAAGAAATCGGCTTCTTTAGCATGGGGCAAGCCTCATTATGATGGTGGGCTTGAAATCACTGGCTATGTGGTAGAGCATCAAAAAGTAGGAGATGATGCCTGGATCAAAGACACAACAGGAAC
AGCCCTCAGAATCACTCAGTTTGTGGTTCCTGATCTTCAGACTAAGGAAAAATACAATTTTAGAATTAGTGCCATCAACGATGCAGGTGTGGGGGAACCAGCTGTGATTCCAAATGTTGAAATTGTAGAGAAGGAAGTGGCTCCTGATTTTGAGCTAGATGCTGAGCTGCGAAGAACACTTGT
GGTTAGGGCAGGGCTCAGTATTAGGATTTTTGTGCCAATTAAAGGTCGTCCTGCACCTGAAGTGACATGGACCAAAGATAATATCAACCTGAAGCACCGAGCCAACATCGAAAACACAGAGTCATTTACTCTTCTCATTATTCCAGAATGTAACAGATATGACACTGGTAAATTTGTGATGACC
ATCGAAAACCCAGCTGGGAAGAAAAGCGGCTTCGTTAACGTCAGAGTCCTAGACACACCAGGCCCTGTCCTTAACCTAAGGCCTACAGACATCACAAAGGACAGTGTCACCCTACACTGGGACCTACCTCTGATAGATGGGGGCTCACGTATAACAAATTATATTGTGGAGAAACGTGAAGC
AACCAGGAAATCCTACTCCACAGTCACTACTAAGTGCCATAAATGTACATATAAAGTGACTGGCTTGACAGAAGGCTGTGAATATTTCTTCAGAGTGATGGCAGAAAATGAATATGGGGTTGGTGAGCCAACGGAAACCACAGAACCTGTGAGAGCCTCTGAGGCACCGTTACCCCCAGATA
GCCTTAACATTATGGACATTACCAAGAACACAGTCAGCCTGGCGTGGCCTAAGCCAAGGCATGATGGTGGGAGCAAGATATCACTGGCTATGGCGGCCGCcATGGTGAGCAAGGGCGAGGCAGTGATCAAGGAGTTCATGCGGTTCAAGGTGCACATGGAGGGCTCCATGAACGGCCACG
AGTTCGAGATCGAGGGCGAGGGCGAGGGaCGCCCCTACGAGGGCACCCAGACCGCCAAGCTGAAGGTGACCAAGGGTGGCCCCCTGCCCTTCTCCTGGGACATCCTGTCCCCTCAGTTCATGTACGGCTCCAGGGCCTTCACCAAGCACCCCGCCGACATCCCCGACTACTATAAGCAGTCC
TTCCCCGAGGGCTTCAAGTGGGAGCGCGTGATGAACTTCGAGGACGGCGGCGCCGTGACCGTGACCCAGGACACCTCCCTGGAGGACGGCACCCTGATCTACAAGGTGAAGCTCCGCGGCACCAACTTCCCTCCTGACGGCCCCGTAATGCAGAAGAAaACAATGGGCTGGGAAGCGTCC
ACCGAGCGGTTGTACCCCGAGGACGGCGTGCTGAAGGGCGACATTAAGATGGCCCTGCGCCTGAAGGACGGCGGaCGCTACCTGGCGGACTTCAAGACCACCTACAAGGCCAAGAAGCCCGTGCAGATGCCCGGCGCCTACAACGTCGACCGCAAGTTGGACATCACCTCCCACAACGAG
GACTACACCGTGGTGGAACAGTACGAACGCTCCGAGGGtCGCCACTCCACCGGtGGtATGGACGAGCTGTACAAGTAGGGCGCGCCCCAGTGGACCCACATCTCAACAGTGAAAGGGTTAGAATGTGTGGTGAGGAACCTAACCGAAGGAGAGGAATATACCTTCCAAGTGATGGCGGTA
AACAGTGCAGGCAGGAGTGCCCCTAGAGAGAGCAGACCTGTCATTGTCAAAGAGCAGACGATGCTCCCAGAGCTGGATCTCCGTGGCATCTATCAGAAGCTAGTCATTGCCAGAGCTGGTGACAACATCAAAGTTGAAATCCCAGTACTTGGTCGACCCAAGCCCACAGTCACATGGAAAA
AGGGAGACCAAATCCTTAAACAGACACAGAGAGTTAATGTTGAAAACACAGCAACGTCAACCATCTTAAATATCAATGAGTGTGTCAGAAGTGACAGTGGACCCTATCCATTAACAGCAAAGAACACTGTAGGAGAGGTTGGTGACGTTATAACCATTCAAGTCCATGATATTCCAGGACCA
CCTACTGGACCCATTAAATTTGATGAAGTTTCATCTGACTTTGTAACCTTCTCTTGGGAGCCACCAGAGAATGATGGAGGTGTACCAATAAGCAACTATGTAGTAGAGATGAGACAGACAGACAGTACTACATGGGTGGAGTTAGCAACAACTGTTATACGTACTACCTATAAAGCAACTCGTC
TGACCACAGGAGTGGAGTATCAATTCCGTGTAAGGGCTCAAAACAGATATGGAGTTGGACCGGGCATCACATCTGCATCTGTAGTTGCAAACTATCCATTCAAGGTGCCCGGGCCTCCCGGTACCCCTCAGGTTACTGCAGTCACCAAAGACTCCATGACAATCAGCTGGCATGAACCACTTT
CTGATGGTGGAAGCCCCATTTTAGGATATCACATTGAAAGGAAAGAACGGAATGGCATCCTCTGGCAGACTGTGAGCAAAGCATTAGTACCAGGCAACATTTTCAAATCAACTGGACTTACCGATGGCATTGCTTATGAATTCCGAGTAATTGCAGAAAACATGGCAGGCAAGAGCGCGATC
GCACATGGGCTAGCGGACTTTGTACACTGGATACTAGTCCCATTACCCTGGTAGATAAGTAGCATGGCGGGTTAATCATTAACTACAAGGAACCCCTAGTGATGGAGTTGGCCACTCCCTCTCTGCGCGCTCGCTCGCTCACTGAGGCCGGGCGACCAAAGGTCGCCCGACGCCCGGGCTTTG
CCCGGGCGGCCTCAGTGAGCGAGCGAGCGCGCAG

AAV-HDR-TTN.P22582fs-mScarlet - inserts mScarlet after truncation caused by P22582fs mutation.
CTGCGCGCTCGCTCGCTCACTGAGGCCGCCCGGGCAAAGCCCGGGCGTCGGGCGACCTTTGGTCGCCCGGCCTCAGTGAGCGAGCGAGCGCGCAGAGAGGGAGTGGCCAACTCCATCACTAGGGGTTCCTTGTAGTTAATGATTAACCCGCCATGCTACTTATCTACCAGGGTAATGGGCT
CGAGGAGGGCCTATTTCCCATGATTCCTTCATATTTGCATATACGATACAAGGCTGTTAGAGAGATAATTGGAATTAATTTGACTGTAAACACAAAGATATTAGTACAAAATACGTGACGTAGAAAGTAATAATTTCTTGGGTAGTTTGCAGTTTTAAAATTATGTTTTAAAATGGACTATCATAT
GCTTACCGTAACTTGAAAGTATTTCGATTTCTTGGCTTTATATATCTTGTGGAAAGGACGAAACACCGGAAGTTACAACTATCACCAGTTTTAGAGCTAGAAATAGCAAGTTAAAATAAGGCTAGTCCGTTATCAACTTGAAAAAGTGGCACCGAGTCGGTGCTTTTTTGGTACCATTGTTCCTG
CAGGTGAATTCCGAGTAATTGCAGAAAACATGGCAGGCAAGAGCAAGCCAAGCAAGCCATCTGAACCAATGTTTGCTTTGGATCCCATTGACCCACCCGGGAAACCAGTTCCTCTAAACATTACAAGACACACAGTGGCACTGAAGTGGGCTAAGCCTGAATATACCGGAGGCTTTAAAATT
ACTAGCTATGTGGTTGAAAAGAGGGACCTTCCTAATGGACGATGGCTGAAGGCCAACTTCAGCAACATTTTAGAGAATGAATTTACGGTCAGCGGCCTAACTGAAGATGCTGCATACGAGTTCCGTGTGATTGCCAAAAATGCTGCCGGTGCCATCAGTCCACCGTCTGAGCCATCAGATGCC
ATCACATGCAGGGACGACCTGGAGGCACCAAGAATAATGGTGGATGTTAGATTTAAGGATACCATCACATTAAAAGCTGGCGAAGCATTCAAGTTGGAAGCTGATGTTTCAGGTCGCCCACCTCCAACAATGGAATGGACTAAAGATGGAAAGGAGCTTGAAGGCACAGGGAAATTAGAA
ATAAAAATTGCAGACTTCTCCACTCATCTGATAAACAAGGATTCCTCAAGAACGGATAGTGGGGCTTACATCCTGACAGCAACCAATCCTGGTGGCTTTGCCAAACACATTTTCAATGTCAAAGTTCTTGATAGACCGGGACCACCTGAGGGACCTTTGGCTGTGTCTGATGTGACATCAGAGA
AGTGTGTGTTGTCATGGTTGCCTCCACTGGACGATGGAGGTGCCAAAATTGATCATTACATCGTACAGAAACGTGAAACCAGCAGGTTGGCATGGACAAATGTTGCCACAGAAGTCCAAGTAACAAAGCTGAAGGTTACTAAGCTCCTGAAAGGGAATGAATACATATTCCGTGTCATGGCA
GTAAATAAATACGGGGTTGGAGAGCCACTTGAATCAGAACCTGTGCTTGCAGTGGATCCATATGGACCACCGACCCACCCAAGAACCCGGAAGATGGTGAGCAAGGGCGAGGCAGTGATCAAGGAGTTCATGCGGTTCAAGGTGCACATGGAGGGCTCCATGAACGGCCACGAGTTCGAG
ATCGAGGGCGAGGGCGAGGGaCGCCCCTACGAGGGCACCCAGACCGCCAAGCTGAAGGTGACCAAGGGTGGCCCCCTGCCCTTCTCCTGGGACATCCTGTCCCCTCAGTTCATGTACGGCTCCAGGGCCTTCACCAAGCACCCCGCCGACATCCCCGACTACTATAAGCAGTCCTTCCCCGA
GGGCTTCAAGTGGGAGCGCGTGATGAACTTCGAGGACGGCGGCGCCGTGACCGTGACCCAGGACACCTCCCTGGAGGACGGCACCCTGATCTACAAGGTGAAGCTCCGCGGCACCAACTTCCCTCCTGACGGCCCCGTAATGCAGAAGAAaACAATGGGCTGGGAAGCGTCCACCGAGCG
GTTGTACCCCGAGGACGGCGTGCTGAAGGGCGACATTAAGATGGCCCTGCGCCTGAAGGACGGCGGaCGCTACCTGGCGGACTTCAAGACCACCTACAAGGCCAAGAAGCCCGTGCAGATGCCCGGCGCCTACAACGTCGACCGCAAGTTGGACATCACCTCCCACAACGAGGACTACAC
CGTGGTGGAACAGTACGAACGCTCCGAGGGtCGCCACTCCACCGGtGGtATGGACGAGCTGTACAAGTAGCTATCACCAAGGACTCCATGGTCGTCTGCTGGGGGCATCCTGACTCTGATGGTGGAAGTGAAATTATCAACTACATTGTGGAGCGGCGTGATAAAGCTGGCCAGCGCTGGGT
AAAATGCAACAAAAAAGCTCTGACTGACTTAAGATTTAAAGTATCTGGGCTGACAGAAGGACATGAATACGAGTTCAGAATTATGGCAGAAAACGCAGCTGGAATCAGTGCTCCGAGTGCTACCAGCCCGTTTTATAAAGCTTGCGACACTGTGTTTAAACCTGGCCCACCGGGTAACCCAC
GTGTCCTTGATACAAGCAGGTCGTCCATTTCAATTGCGTGGAATAAACCCATTTATGATGGAGGTTCAGAAATCACTGGGTATATGGTTGAGATTGCCCTGCCTGAAGAAGATGAATGGCAGGTTGTCACTCCACCAGCTGGGCTCAAGGCAACTTCCTATACCATCACCAGCCTCATAGAGA
ATCAGGAATATAAAATCCGCATTTATGCCATGAATTCTGAGGGACTTGGGGAACCTGCCCTTGTTCCCGGAACTCCAAAGGCAGAAGAAAGAATGTTGCCTCCAGAGATAGAACTGGATGCTGACTTACGCAAAGTTGTCACTATACGAGCCTGCTGCACCTTGAGGCTCTTTGTTCCCATCAA
AGGGAGACCAGCACCTGAGGTCAAGTGGGCCCGAGAGCATGGAGAATCTCTGGACAAGGCTAGCATTGAATCCACAAGCTCTTACACCCTGCTTGTTGTTGGAAATGTTAACAGATTCGACAGTGGCAAGTACATACTAACGGTAGAAAACAGCTCTGGCAGCAAGTCGGCGTTTGTCAAT
GTTAGAGTGCTAGATACGCCAGGCCCTCCACAGAATCTAAAGATAAAGGAGGTCACAAAGACATCTGTCACATTGACGTGGGAGCCTCCTCTCCTGGATGGAGGCTCAAAAATAAAGAACTATATTGTTGAGAAGCGAGAGTCCACAAGAAAGGCATAGCGATCGCACATGGGCTAGCGGA
CTTTGTACACTGGATACTAGTCCCATTACCCTGGTAGATAAGTAGCATGGCGGGTTAATCATTAACTACAAGGAACCCCTAGTGATGGAGTTGGCCACTCCCTCTCTGCGCGCTCGCTCGCTCACTGAGGCCGGGCGACCAAAGGTCGCCCGACGCCCGGGCTTTGCCCGGGCGGCCTCAGTG
AGCGAGCGAGCGCGCAG

AAV-HDR-mScarlet-PLN-WT - inserts mScarlet at the N-terminus of wildtype Pln.
CTGCGCGCTCGCTCGCTCACTGAGGCCGCCCGGGCAAAGCCCGGGCGTCGGGCGACCTTTGGTCGCCCGGCCTCAGTGAGCGAGCGAGCGCGCAGAGAGGGAGTGGCCAACTCCATCACTAGGGGTTCCTTGTAGTTAATGATTAACCCGCCATGCTACTTATCTACCAGGGTAATGGGCT
CGAGGAGGGCCTATTTCCCATGATTCCTTCATATTTGCATATACGATACAAGGCTGTTAGAGAGATAATTGGAATTAATTTGACTGTAAACACAAAGATATTAGTACAAAATACGTGACGTAGAAAGTAATAATTTCTTGGGTAGTTTGCAGTTTTAAAATTATGTTTTAAAATGGACTATCATAT
GCTTACCGTAACTTGAAAGTATTTCGATTTCTTGGCTTTATATATCTTGTGGAAAGGACGAAACACCGGGAGACAAGTTTTTCTAAGGTTTTAGAGCTAGAAATAGCAAGTTAAAATAAGGCTAGTCCGTTATCAACTTGAAAAAGTGGCACCGAGTCGGTGCTTTTTTGGTACCATTGTTCCTG
CAGGATACATTGTATTTCCAGCACTCTGGTACAAAAATCAGAAAGATTAAACTTCAAATTAGTCTGTAAGTAAAGAGGCTCTGTGTCTCCAGTAAACACCCAATGAACATATACAGTCATCTTTTTAAACCTGTAGCTAATTTTCTGTTAACACGCTTTATTTTGTTGTATTTACTATCAAAGCTTTG
AACCTTACATATTCAGCCATAGTTTAGAAGTTTTGAAAGAAGGCCAGACACAAACCCACCCCTGGTAGCCAAGATTAGCTGAAAACTGGCACTGTGATAGTTTAAAACGATAGTCCTTACTGTGCCTTCTGAGTTTGGAAGTCTTTAGTACTTAACATCTTCTTAAAGTGTTTAAAATAACATTCA
CCAAGAGGCTGGATCTGGGAGGAGTCCGGAGGATGTATATATGATCAAATACACTGAATGATTCTACAAATCTATAAATCATAAAAGAGAATGGCAAGTGGCTAGAATCTGCTTTTTATTTCATATTTTTCACTTAACTATTTTATTTTGGACTAAAACTAGGATCCAAGATATGTTACTATATGC
ATTTCTTTTTACAAGATTATTTCTGAAACATTAATAGTAAATGTTAAAATCAATTGTGTTTACAGAGAAACAGAGCTTTAAAAAAAGTAACCAAGAAAATAGCCCCAAAGAAGCACCTCATAAATCAAGTACTAGCCGCCAAAGCCGTGGCTCTCCCCGCTCACCCAGGACAGTGAGACGCTCA
CACATGAGAGACAGAAGAGAGGCCTTGGGGACTGGAGAAGGGGATGGGATTTAAAATACAGCTGAGGAACGAGCTGCTGCAAAGCTGTGCATATCTACCCAGAGAACGGGTTTAGGTATGAACCTAATCTGTTGACTATTTTTTACATCCCAGGTCTCCTAAAAGAAGACAGCTCACATTTG
GCTGCCTGTTGTCAACTTTTTATCTTTCTCTTGACAACTTAGAAAAACTTGTCTCCCTACTTTTGCCTTCCTGGCATAgccaccATGGTGAGCAAGGGCGAGGCAGTGATCAAGGAGTTCATGCGGTTCAAGGTGCACATGGAGGGCTCCATGAACGGCCACGAGTTCGAGATCGAGGGCGAGG
GCGAGGGaCGCCCCTACGAGGGCACCCAGACCGCCAAGCTGAAGGTGACCAAGGGTGGCCCCCTGCCCTTCTCCTGGGACATCCTGTCCCCTCAGTTCATGTACGGCTCCAGGGCCTTCACCAAGCACCCCGCCGACATCCCCGACTACTATAAGCAGTCCTTCCCCGAGGGCTTCAAGTGGG
AGCGCGTGATGAACTTCGAGGACGGCGGCGCCGTGACCGTGACCCAGGACACCTCCCTGGAGGACGGCACCCTGATCTACAAGGTGAAGCTCCGCGGCACCAACTTCCCTCCTGACGGCCCCGTAATGCAGAAGAAaACAATGGGCTGGGAAGCGTCCACCGAGCGGTTGTACCCCGAGG
ACGGCGTGCTGAAGGGCGACATTAAGATGGCCCTGCGCCTGAAGGACGGCGGaCGCTACCTGGCGGACTTCAAGACCACCTACAAGGCCAAGAAGCCCGTGCAGATGCCCGGCGCCTACAACGTCGACCGCAAGTTGGACATCACCTCCCACAACGAGGACTACACCGTGGTGGAACAGT
ACGAACGCTCCGAGGGtCGCCACTCCACCGGtGGtATGGACGAGCTGTACAAGATGGAAAAAGTGCAATACCTCACTCGCTCGGCTATCAGGAGAGCCTCCACTATTGAAATGCCTCAGCAAGCACGTCAGAATCTCCAGAACCTATTTATCAATTTCTGCCTCATCTTGATATGTCTGCTGCTG
ATCTGCATCATTGTGATGCTTCTGTGAAGAGCTGCCGCCACTCCAGACCTGCAACATGCCAACTCAGCTTAAAGCCGAGCACTCCGTCATGGGGGAAGCAACGCACCGCTGCCTTCCGGCTGAGAACAGCTTTGTGAGGGTCACGATTAAGAGTGAAACTAATTGTTAGCCAGTGTATTCATC
TGCTGGATCTTGTAAACAAACTTTACAGTGGCTAAGTGTGCATAATGTAGCTGCCAATTTCCTCAACATGGCTCATAAATTTCTTTCCTAAATATTTCTGACAATGAAGTATGAGTTTTAATTTTGAAACACCACTACCAAAAAAGTTCACTTTCTATATCCAACTCTAGCAAGGTCACTTTATAGCT
CCAGTCTGGGGAATAAATGGAATATATACTGCATTGCCACTACCTTTTTTATAGGAACTAAGTGTTCTTCAGTTCTCCACAAAGTAGAAAATAGTCATTTAAATGAAGTGTTCTTATTGAAAATAGCTCACTGGGTCCTCACATAGATGACACAATGATGACGATGATGATGATGACGACGATGA
TGATGATGACGACGATTCAAATCTCTTGGTCACTACTGTCAGAACAAATACCTTGAAAAAATGTTGTGCCCTTTTTCTACACACTAGTATTTTTAAGCTTATGAGAGTTAAAGGGGGAAATGTAAGAACATATTCTCATATAAAATTATTCACTAATTTTGCTCAAGGGCTGTGTACATGTACATG
TACATTAACCACAAATCTGCTCTCTCTAGGGTTTGATCAACAAGCAGGAACATCTTGCTGTGTTGGCTGTGATAATCACTAAAGATCCAGAGTCTCACCTGAAGCTTGTCGCTCTCATGTATCTCCTTGGCGATCGCACATGGGCTAGCGGACTTTGTACACTGGATACTAGTCCCATTACCCTGG
TAGATAAGTAGCATGGCGGGTTAATCATTAACTACAAGGAACCCCTAGTGATGGAGTTGGCCACTCCCTCTCTGCGCGCTCGCTCGCTCACTGAGGCCGGGCGACCAAAGGTCGCCCGACGCCCGGGCTTTGCCCGGGCGGCCTCAGTGAGCGAGCGAGCGCGCAG

AAV-HDR-mScarlet-PLN-R14Del - inserts mScarlet at the N-terminus of R14Del Pln.

CTGCGCGCTCGCTCGCTCACTGAGGCCGCCCGGGCAAAGCCCGGGCGTCGGGCGACCTTTGGTCGCCCGGCCTCAGTGAGCGAGCGAGCGCGCAGAGAGGGAGTGGCCAACTCCATCACTAGGGGTTCCTTGTAGTTAATGATTAACCCGCCATGCTACTTATCTACCAGGGTAATGGGCTCGAGGAGGG
CCTATTTCCCATGATTCCTTCATATTTGCATATACGATACAAGGCTGTTAGAGAGATAATTGGAATTAATTTGACTGTAAACACAAAGATATTAGTACAAAATACGTGACGTAGAAAGTAATAATTTCTTGGGTAGTTTGCAGTTTTAAAATTATGTTTTAAAATGGACTATCATATGCTTACCGTAACTTGAAA
GTATTTCGATTTCTTGGCTTTATATATCTTGTGGAAAGGACGAAACACCGGGAGACAAGTTTTTCTAAGGTTTTAGAGCTAGAAATAGCAAGTTAAAATAAGGCTAGTCCGTTATCAACTTGAAAAAGTGGCACCGAGTCGGTGCTTTTTTGGTACCATTGTTCCTGCAGGATACATTGTATTTCCAGCACTCT
GGTACAAAAATCAGAAAGATTAAACTTCAAATTAGTCTGTAAGTAAAGAGGCTCTGTGTCTCCAGTAAACACCCAATGAACATATACAGTCATCTTTTTAAACCTGTAGCTAATTTTCTGTTAACACGCTTTATTTTGTTGTATTTACTATCAAAGCTTTGAACCTTACATATTCAGCCATAGTTTAGAAGTTTTGA
AAGAAGGCCAGACACAAACCCACCCCTGGTAGCCAAGATTAGCTGAAAACTGGCACTGTGATAGTTTAAAACGATAGTCCTTACTGTGCCTTCTGAGTTTGGAAGTCTTTAGTACTTAACATCTTCTTAAAGTGTTTAAAATAACATTCACCAAGAGGCTGGATCTGGGAGGAGTCCGGAGGATGTATATATG
ATCAAATACACTGAATGATTCTACAAATCTATAAATCATAAAAGAGAATGGCAAGTGGCTAGAATCTGCTTTTTATTTCATATTTTTCACTTAACTATTTTATTTTGGACTAAAACTAGGATCCAAGATATGTTACTATATGCATTTCTTTTTACAAGATTATTTCTGAAACATTAATAGTAAATGTTAAAATCAAT
TGTGTTTACAGAGAAACAGAGCTTTAAAAAAAGTAACCAAGAAAATAGCCCCAAAGAAGCACCTCATAAATCAAGTACTAGCCGCCAAAGCCGTGGCTCTCCCCGCTCACCCAGGACAGTGAGACGCTCACACATGAGAGACAGAAGAGAGGCCTTGGGGACTGGAGAAGGGGATGGGATTTAAAATAC
AGCTGAGGAACGAGCTGCTGCAAAGCTGTGCATATCTACCCAGAGAACGGGTTTAGGTATGAACCTAATCTGTTGACTATTTTTTACATCCCAGGTCTCCTAAAAGAAGACAGCTCACATTTGGCTGCCTGTTGTCAACTTTTTATCTTTCTCTTGACAACTTAGAAAAACTTGTCTCCCTACTTTTGCCTTCCTG
GCATAgccaccATGGTGAGCAAGGGCGAGGCAGTGATCAAGGAGTTCATGCGGTTCAAGGTGCACATGGAGGGCTCCATGAACGGCCACGAGTTCGAGATCGAGGGCGAGGGCGAGGGaCGCCCCTACGAGGGCACCCAGACCGCCAAGCTGAAGGTGACCAAGGGTGGCCCCCTGCCCTTCTCCTGGG
ACATCCTGTCCCCTCAGTTCATGTACGGCTCCAGGGCCTTCACCAAGCACCCCGCCGACATCCCCGACTACTATAAGCAGTCCTTCCCCGAGGGCTTCAAGTGGGAGCGCGTGATGAACTTCGAGGACGGCGGCGCCGTGACCGTGACCCAGGACACCTCCCTGGAGGACGGCACCCTGATCTACAAGGTGA
AGCTCCGCGGCACCAACTTCCCTCCTGACGGCCCCGTAATGCAGAAGAAaACAATGGGCTGGGAAGCGTCCACCGAGCGGTTGTACCCCGAGGACGGCGTGCTGAAGGGCGACATTAAGATGGCCCTGCGCCTGAAGGACGGCGGaCGCTACCTGGCGGACTTCAAGACCACCTACAAGGCCAAGAAGC
CCGTGCAGATGCCCGGCGCCTACAACGTCGACCGCAAGTTGGACATCACCTCCCACAACGAGGACTACACCGTGGTGGAACAGTACGAACGCTCCGAGGGtCGCCACTCCACCGGtGGtATGGACGAGCTGTACAAGATGGAAAAAGTGCAATACCTCACTCGCTCGGCTATCAGGGCCTCCACTATTGAAA
TGCCTCAGCAAGCACGTCAGAATCTCCAGAACCTATTTATCAATTTCTGCCTCATCTTGATATGTCTGCTGCTGATCTGCATCATTGTGATGCTTCTGTGAAGAGCTGCCGCCACTCCAGACCTGCAACATGCCAACTCAGCTTAAAGCCGAGCACTCCGTCATGGGGGAAGCAACGCACCGCTGCCTTCCGGCT
GAGAACAGCTTTGTGAGGGTCACGATTAAGAGTGAAACTAATTGTTAGCCAGTGTATTCATCTGCTGGATCTTGTAAACAAACTTTACAGTGGCTAAGTGTGCATAATGTAGCTGCCAATTTCCTCAACATGGCTCATAAATTTCTTTCCTAAATATTTCTGACAATGAAGTATGAGTTTTAATTTTGAAACACC
ACTACCAAAAAAGTTCACTTTCTATATCCAACTCTAGCAAGGTCACTTTATAGCTCCAGTCTGGGGAATAAATGGAATATATACTGCATTGCCACTACCTTTTTTATAGGAACTAAGTGTTCTTCAGTTCTCCACAAAGTAGAAAATAGTCATTTAAATGAAGTGTTCTTATTGAAAATAGCTCACTGGGTCCTCA
CATAGATGACACAATGATGACGATGATGATGATGACGACGATGATGATGATGACGACGATTCAAATCTCTTGGTCACTACTGTCAGAACAAATACCTTGAAAAAATGTTGTGCCCTTTTTCTACACACTAGTATTTTTAAGCTTATGAGAGTTAAAGGGGGAAATGTAAGAACATATTCTCATATAAAATTATT
CACTAATTTTGCTCAAGGGCTGTGTACATGTACATGTACATTAACCACAAATCTGCTCTCTCTAGGGTTTGATCAACAAGCAGGAACATCTTGCTGTGTTGGCTGTGATAATCACTAAAGATCCAGAGTCTCACCTGAAGCTTGTCGCTCTCATGTATCTCCTTGGCGATCGCACATGGGCTAGCGGACTTTGTA
CACTGGATACTAGTCCCATTACCCTGGTAGATAAGTAGCATGGCGGGTTAATCATTAACTACAAGGAACCCCTAGTGATGGAGTTGGCCACTCCCTCTCTGCGCGCTCGCTCGCTCACTGAGGCCGGGCGACCAAAGGTCGCCCGACGCCCGGGCTTTGCCCGGGCGGCCTCAGTGAGCGAGCGAGCGCGC
AG

AAV-HDR-SNAP-PLN-WT - inserts SNAP-tag at the N-terminus of wildtype Pln.

CTGCGCGCTCGCTCGCTCACTGAGGCCGCCCGGGCAAAGCCCGGGCGTCGGGCGACCTTTGGTCGCCCGGCCTCAGTGAGCGAGCGAGCGCGCAGAGAGGGAGTGGCCAACTCCATCACTAGGGGTTCCTTGTAGTTAATGATTAACCCGCCATGCTACTTATCTACCAGGGTAATGGGCTCGAGGAGGG
CCTATTTCCCATGATTCCTTCATATTTGCATATACGATACAAGGCTGTTAGAGAGATAATTGGAATTAATTTGACTGTAAACACAAAGATATTAGTACAAAATACGTGACGTAGAAAGTAATAATTTCTTGGGTAGTTTGCAGTTTTAAAATTATGTTTTAAAATGGACTATCATATGCTTACCGTAACTTGAAA
GTATTTCGATTTCTTGGCTTTATATATCTTGTGGAAAGGACGAAACACCGGGAGACAAGTTTTTCTAAGGTTTTAGAGCTAGAAATAGCAAGTTAAAATAAGGCTAGTCCGTTATCAACTTGAAAAAGTGGCACCGAGTCGGTGCTTTTTTGGTACCATTGTTCCTGCAGGATACATTGTATTTCCAGCACTCT
GGTACAAAAATCAGAAAGATTAAACTTCAAATTAGTCTGTAAGTAAAGAGGCTCTGTGTCTCCAGTAAACACCCAATGAACATATACAGTCATCTTTTTAAACCTGTAGCTAATTTTCTGTTAACACGCTTTATTTTGTTGTATTTACTATCAAAGCTTTGAACCTTACATATTCAGCCATAGTTTAGAAGTTTTGA
AAGAAGGCCAGACACAAACCCACCCCTGGTAGCCAAGATTAGCTGAAAACTGGCACTGTGATAGTTTAAAACGATAGTCCTTACTGTGCCTTCTGAGTTTGGAAGTCTTTAGTACTTAACATCTTCTTAAAGTGTTTAAAATAACATTCACCAAGAGGCTGGATCTGGGAGGAGTCCGGAGGATGTATATATG
ATCAAATACACTGAATGATTCTACAAATCTATAAATCATAAAAGAGAATGGCAAGTGGCTAGAATCTGCTTTTTATTTCATATTTTTCACTTAACTATTTTATTTTGGACTAAAACTAGGATCCAAGATATGTTACTATATGCATTTCTTTTTACAAGATTATTTCTGAAACATTAATAGTAAATGTTAAAATCAAT
TGTGTTTACAGAGAAACAGAGCTTTAAAAAAAGTAACCAAGAAAATAGCCCCAAAGAAGCACCTCATAAATCAAGTACTAGCCGCCAAAGCCGTGGCTCTCCCCGCTCACCCAGGACAGTGAGACGCTCACACATGAGAGACAGAAGAGAGGCCTTGGGGACTGGAGAAGGGGATGGGATTTAAAATAC
AGCTGAGGAACGAGCTGCTGCAAAGCTGTGCATATCTACCCAGAGAACGGGTTTAGGTATGAACCTAATCTGTTGACTATTTTTTACATCCCAGGTCTCCTAAAAGAAGACAGCTCACATTTGGCTGCCTGTTGTCAACTTTTTATCTTTCTCTTGACAACTTAGAAAAACTTGTCTCCCTACTTTTGCCTTCCTG
GCATAgccaccatggacaaagactgcgaaatgaagcgcaccaccctggatagccctctgggcaagctggaactgtctgggtgcgaacagggcctgcaccgtatcatcttcctgggcaaaggaacatctgccgccgacgccgtggaagtgcctgccccagccgccgtgctgggcggaccagagccactgatgcaggccaccgcctggctcaacgcctactttcaccagcctgaggccatcgagg
agttccctgtgccagccctgcaccacccagtgttccagcaggagagctttacccgccaggtgctgtggaaactgctgaaagtggtgaagttcggagaggtcatcagctacagccacctggccgccctggccggcaatcccgccgccaccgccgccgtgaaaaccgccctgagcggaaatcccgtgcccattctgatcccctgccaccgggtggtgcagggcgacctggacgtggggggctacgagg
gcgggctcgccgtgaaagagtggctgctggcccacgagggccacagactgggcaagcctgggctgggtATGGAAAAAGTGCAATACCTCACTCGCTCGGCTATCAGGAGAGCCTCCACTATTGAAATGCCTCAGCAAGCACGTCAGAATCTCCAGAACCTATTTATCAATTTCTGCCTCATCTTGATATGTCTGCTGCTGATCTGCAT
CATTGTGATGCTTCTGTGAAGAGCTGCCGCCACTCCAGACCTGCAACATGCCAACTCAGCTTAAAGCCGAGCACTCCGTCATGGGGGAAGCAACGCACCGCTGCCTTCCGGCTGAGAACAGCTTTGTGAGGGTCACGATTAAGAGTGAAACTAATTGTTAGCCAGTGTATTCATCTGCTGGATCTTGTAAACA
AACTTTACAGTGGCTAAGTGTGCATAATGTAGCTGCCAATTTCCTCAACATGGCTCATAAATTTCTTTCCTAAATATTTCTGACAATGAAGTATGAGTTTTAATTTTGAAACACCACTACCAAAAAAGTTCACTTTCTATATCCAACTCTAGCAAGGTCACTTTATAGCTCCAGTCTGGGGAATAAATGGAATATA
TACTGCATTGCCACTACCTTTTTTATAGGAACTAAGTGTTCTTCAGTTCTCCACAAAGTAGAAAATAGTCATTTAAATGAAGTGTTCTTATTGAAAATAGCTCACTGGGTCCTCACATAGATGACACAATGATGACGATGATGATGATGACGACGATGATGATGATGACGACGATTCAAATCTCTTGGTCACTA
CTGTCAGAACAAATACCTTGAAAAAATGTTGTGCCCTTTTTCTACACACTAGTATTTTTAAGCTTATGAGAGTTAAAGGGGGAAATGTAAGAACATATTCTCATATAAAATTATTCACTAATTTTGCTCAAGGGCTGTGTACATGTACATGTACATTAACCACAAATCTGCTCTCTCTAGGGTTTGATCAACAAG
CAGGAACATCTTGCTGTGTTGGCTGTGATAATCACTAAAGATCCAGAGTCTCACCTGAAGCTTGTCGCTCTCATGTATCTCCTTGGCGATCGCACATGGGCTAGCGGACTTTGTACACTGGATACTAGTCCCATTACCCTGGTAGATAAGTAGCATGGCGGGTTAATCATTAACTACAAGGAACCCCTAGTGAT
GGAGTTGGCCACTCCCTCTCTGCGCGCTCGCTCGCTCACTGAGGCCGGGCGACCAAAGGTCGCCCGACGCCCGGGCTTTGCCCGGGCGGCCTCAGTGAGCGAGCGAGCGCGCAG

AAV-HDR-SNAP-PLN-R14Del - inserts SNAP-tag at the N-terminus of R14Del Pln.

CTGCGCGCTCGCTCGCTCACTGAGGCCGCCCGGGCAAAGCCCGGGCGTCGGGCGACCTTTGGTCGCCCGGCCTCAGTGAGCGAGCGAGCGCGCAGAGAGGGAGTGGCCAACTCCATCACTAGGGGTTCCTTGTAGTTAATGATTAACCCGCCATGCTACTTATCTACCAGGGTAATGGGCTCGAGGAGGG
CCTATTTCCCATGATTCCTTCATATTTGCATATACGATACAAGGCTGTTAGAGAGATAATTGGAATTAATTTGACTGTAAACACAAAGATATTAGTACAAAATACGTGACGTAGAAAGTAATAATTTCTTGGGTAGTTTGCAGTTTTAAAATTATGTTTTAAAATGGACTATCATATGCTTACCGTAACTTGAAA
GTATTTCGATTTCTTGGCTTTATATATCTTGTGGAAAGGACGAAACACCGGGAGACAAGTTTTTCTAAGGTTTTAGAGCTAGAAATAGCAAGTTAAAATAAGGCTAGTCCGTTATCAACTTGAAAAAGTGGCACCGAGTCGGTGCTTTTTTGGTACCATTGTTCCTGCAGGATACATTGTATTTCCAGCACTCT
GGTACAAAAATCAGAAAGATTAAACTTCAAATTAGTCTGTAAGTAAAGAGGCTCTGTGTCTCCAGTAAACACCCAATGAACATATACAGTCATCTTTTTAAACCTGTAGCTAATTTTCTGTTAACACGCTTTATTTTGTTGTATTTACTATCAAAGCTTTGAACCTTACATATTCAGCCATAGTTTAGAAGTTTTGA
AAGAAGGCCAGACACAAACCCACCCCTGGTAGCCAAGATTAGCTGAAAACTGGCACTGTGATAGTTTAAAACGATAGTCCTTACTGTGCCTTCTGAGTTTGGAAGTCTTTAGTACTTAACATCTTCTTAAAGTGTTTAAAATAACATTCACCAAGAGGCTGGATCTGGGAGGAGTCCGGAGGATGTATATATG
ATCAAATACACTGAATGATTCTACAAATCTATAAATCATAAAAGAGAATGGCAAGTGGCTAGAATCTGCTTTTTATTTCATATTTTTCACTTAACTATTTTATTTTGGACTAAAACTAGGATCCAAGATATGTTACTATATGCATTTCTTTTTACAAGATTATTTCTGAAACATTAATAGTAAATGTTAAAATCAAT
TGTGTTTACAGAGAAACAGAGCTTTAAAAAAAGTAACCAAGAAAATAGCCCCAAAGAAGCACCTCATAAATCAAGTACTAGCCGCCAAAGCCGTGGCTCTCCCCGCTCACCCAGGACAGTGAGACGCTCACACATGAGAGACAGAAGAGAGGCCTTGGGGACTGGAGAAGGGGATGGGATTTAAAATAC
AGCTGAGGAACGAGCTGCTGCAAAGCTGTGCATATCTACCCAGAGAACGGGTTTAGGTATGAACCTAATCTGTTGACTATTTTTTACATCCCAGGTCTCCTAAAAGAAGACAGCTCACATTTGGCTGCCTGTTGTCAACTTTTTATCTTTCTCTTGACAACTTAGAAAAACTTGTCTCCCTACTTTTGCCTTCCTG
GCATAgccaccatggacaaagactgcgaaatgaagcgcaccaccctggatagccctctgggcaagctggaactgtctgggtgcgaacagggcctgcaccgtatcatcttcctgggcaaaggaacatctgccgccgacgccgtggaagtgcctgccccagccgccgtgctgggcggaccagagccactgatgcaggccaccgcctggctcaacgcctactttcaccagcctgaggccatcgagg
agttccctgtgccagccctgcaccacccagtgttccagcaggagagctttacccgccaggtgctgtggaaactgctgaaagtggtgaagttcggagaggtcatcagctacagccacctggccgccctggccggcaatcccgccgccaccgccgccgtgaaaaccgccctgagcggaaatcccgtgcccattctgatcccctgccaccgggtggtgcagggcgacctggacgtggggggctacgagg
gcgggctcgccgtgaaagagtggctgctggcccacgagggccacagactgggcaagcctgggctgggtATGGAAAAAGTGCAATACCTCACTCGCTCGGCTATCAGGGCCTCCACTATTGAAATGCCTCAGCAAGCACGTCAGAATCTCCAGAACCTATTTATCAATTTCTGCCTCATCTTGATATGTCTGCTGCTGATCTGCATCATT
GTGATGCTTCTGTGAAGAGCTGCCGCCACTCCAGACCTGCAACATGCCAACTCAGCTTAAAGCCGAGCACTCCGTCATGGGGGAAGCAACGCACCGCTGCCTTCCGGCTGAGAACAGCTTTGTGAGGGTCACGATTAAGAGTGAAACTAATTGTTAGCCAGTGTATTCATCTGCTGGATCTTGTAAACAAAC
TTTACAGTGGCTAAGTGTGCATAATGTAGCTGCCAATTTCCTCAACATGGCTCATAAATTTCTTTCCTAAATATTTCTGACAATGAAGTATGAGTTTTAATTTTGAAACACCACTACCAAAAAAGTTCACTTTCTATATCCAACTCTAGCAAGGTCACTTTATAGCTCCAGTCTGGGGAATAAATGGAATATATAC
TGCATTGCCACTACCTTTTTTATAGGAACTAAGTGTTCTTCAGTTCTCCACAAAGTAGAAAATAGTCATTTAAATGAAGTGTTCTTATTGAAAATAGCTCACTGGGTCCTCACATAGATGACACAATGATGACGATGATGATGATGACGACGATGATGATGATGACGACGATTCAAATCTCTTGGTCACTACTG
TCAGAACAAATACCTTGAAAAAATGTTGTGCCCTTTTTCTACACACTAGTATTTTTAAGCTTATGAGAGTTAAAGGGGGAAATGTAAGAACATATTCTCATATAAAATTATTCACTAATTTTGCTCAAGGGCTGTGTACATGTACATGTACATTAACCACAAATCTGCTCTCTCTAGGGTTTGATCAACAAGCAG
GAACATCTTGCTGTGTTGGCTGTGATAATCACTAAAGATCCAGAGTCTCACCTGAAGCTTGTCGCTCTCATGTATCTCCTTGGCGATCGCACATGGGCTAGCGGACTTTGTACACTGGATACTAGTCCCATTACCCTGGTAGATAAGTAGCATGGCGGGTTAATCATTAACTACAAGGAACCCCTAGTGATGGA
GTTGGCCACTCCCTCTCTGCGCGCTCGCTCGCTCACTGAGGCCGGGCGACCAAAGGTCGCCCGACGCCCGGGCTTTGCCCGGGCGGCCTCAGTGAGCGAGCGAGCGCGCAG

AAV-HDR-Tnni1-2xHA-Enh - inserts 2xHA-tag into Tnni1 C-terminus, and enhancer pool into Tnni1 3' UTR. N = enhancer and barcode.

CTGCGCGCTCGCTCGCTCACTGAGGCCGCCCGGGCAAAGCCCGGGCGTCGGGCGACCTTTGGTCGCCCGGCCTCAGTGAGCGAGCGAGCGCGCAGAGAGGGAGTGGCCAACTCCATCACTAGGGGTTCCTTGTAGTTAATGATTAACCCGCCATGCTACTTATCTACCAGGGTAATGGGCTCGAGGAGGG
CCTATTTCCCATGATTCCTTCATATTTGCATATACGATACAAGGCTGTTAGAGAGATAATTGGAATTAATTTGACTGTAAACACAAAGATATTAGTACAAAATACGTGACGTAGAAAGTAATAATTTCTTGGGTAGTTTGCAGTTTTAAAATTATGTTTTAAAATGGACTATCATATGCTTACCGTAACTTGAAA
GTATTTCGATTTCTTGGCTTTATATATCTTGTGGAAAGGACGAAACACCCTCTAAGCACCTCTACTGGGGTTTTAGAGCTAGAAATAGCAAGTTAAAATAAGGCTAGTCCGTTATCAACTTGAAAAAGTGGCACCGAGTCGGTGCTTTTTTGGTACCATTGTTCCTGCAGGGCCCTACTGGGCTCTAAGCACAA
GGTGTCCATGGATCTGCGGGCCAACCTCAAGTCTGTGAAGAAAGAAGACACAGAAAAGGTGAGGGCCCCTCTCCTCAGACCCGCCTACTGGCCTCTATCTGCCACTTCTTACATTGCCTGAGGCGCTCTAGTCAGTGTGTGAATCAGGATATTCTAGGATCCCCTAGGAGGCCAGTGTCAGAAGAGAGACTA
CTGGCTGCTTCCAGTCTGAGGGGACAATGGGATGAGAGAAAGGCTGACTGGGAAATTAAAACTTGCTTCCCATATCAGTTAAGGGGTAACCATTACCCCTGTCACTTCAGCTTCTCGTTGTGGCTATAGGAACATGAGTGCATAGAAATTGGAGAACCGAGTTCAATTAACTTGCCTAGCAAACAACTACTTC
AAATTTGTTGGTCTCACAGAACATGTTTAGAGCTGACAGGCTGTGAGGAGCATGGTGAGAGAGCAATTTATCTGGCCCAGAGAGCTTAATAAAGACAAACAGGCCAAGCCAGTATGCTGACAAAATTGAAAACAAGTTAAATGAATCAGGAGAGGCTTCTTGGAGGAGGTGACCTTGAACCAACTGCAGG
GTGTCCCACAGGAGGGTGTTGTGGATCCTGGAGGAGAGGTTGGCCTGTCATATACTGGGAACCAGTTTTGTGACCCTGTGACAGTTACCTAGACCCTCCCATCCACACAGCCTCATTCTGTGCTCCTGTCTGGCCTATTCATCTTGTATCATGTGCTGGGCAGGGCCATCAGGATTTTTTAGCTCTCTGAGGAG
GTAGATTTTCTCCCATCTACTTTCTCTTGAGCTTGTGGGTCCTTTGTTGCCCCTGCGGCCTGGTGAGGCCCCTGCAGTGCTGACATGTTTGTGGACTGCCCACAGGAGCGGCCAGTCGAGGTAGGAGACTGGAGGAAGAATGTGGAGGCTATGTCTGGCATGGAAGGCCGCAAGAAGATGTTCGACGCTGCT
AAGTCCCCGACCTCCCAGTACCCCTACGACGTGCCCGACTACGCTTACCCTTACGATGTGCCTGATTATGCCTAGGCGGCCGCNNNNNNNNNNNNNNNNNNNNNNNNNNNNNNNNNNNNNNNNNNNNNNNNNNNNNNNNNNNNNNNNNNNNNNNNNNNNNNNNNNNNNNN
NNNNNNNNNNNNNNNNNNNNNNNNNNNNNNNNNNNNNNNNNNNNNNNNNNNNNNNNNNNNNNNNNNNNNNNNNNNNNNNNNNNNNNNNNNNNNNNNNNNNNNNNNNNNNNNNNNNNNNNNNNNNNNNNNNNNNNNNNNNN
NNNNNNNNNNNNNNNNNNNNNNNNNNNNNNNNNNNNNNNNNNNNNNNNNNNNNNNNNNNNNNNNNNNNNNNNNNNNNNNNNNNNNNNNNNNNNNNNNNNNNNNNNNNNNNATCGATAATCGGCCGGCCGTCAGCGATCGCNNNN
NNNNNNGGCGCGCCCAGGTGAGTAGTAGAGGTGCTTAGAGTGTTCTTGGggtccctcagagtaccccagggtactcaggagaagggatgggaggcagatccaatgtgcagaactccagcctgctccagtaccttcagacactgggcacagcatgtctatgccagatctttttgtccccttatcagtaaaacttgggcttgtatgtgatcagtggtcctggaactggcagcat
ctatgtgttcctggttgcaaatgactgaactggaaactggggggaccctagaggaccctgagacaggcaagagtctgagagtacagggttaggtgatgcacaaggtccctccagctctaacgcttttctaactcagtcttttctttcacttgtcacttggtgccatacttggcttccataccctcaaagaagctcccccagtcactactcccagagaaacagcagttaagagatgttcaaacccccccc
ccactccagagaaatagctgcaggaactgctgaagcagtggaattgggccataaaagccctgctgtgggccctaggaggggaggtgtaatagccaggtctacaggccacacagagccaacttcttcacagagcagatgttagctagtgataggtgagggttcacacatgaaggggaagggctgagggtgcaaaggtcagctatgggagggaaccggtctggaagatggccaggacaggttgg
agggaaggagctagagagggaggcagcttgggatctggacagagcagctaagtcccatgggtggtacaagggagaagccccagggactatgggggctgtgtgtgctgtgtgttaatcagggttacctaaacagcacgcatcactgggatctatctgatggcatctcctcttttgtcctgacaggttggcttgctgtatgacactctgtgtcatgctttggtccctttccagccccactcacctatctttct
gctcgtgcctggaacatccctaatatgcGCTCCCTCCCTCCAGCCTATGCGATCGCACATGGGCTAGCGGACTTTGTACACTGGATACTAGTCCCATTACCCTGGTAGATAAGTAGCATGGCGGGTTAATCATTAACTACAAGGAACCCCTAGTGATGGAGTTGGCCACTCCCTCTCTGCGCGCTCGCTCGCTCACTGAG
GCCGGGCGACCAAAGGTCGCCCGACGCCCGGGCTTTGCCCGGGCGGCCTCAGTGAGCGAGCGAGCGCGCAG

AAV-Enh-Prom-mCherry-bc-polyA - MPRA vector carrying barcoded enhancer:promoter pairs, driving expression of mCherry reporter transcript. N = enhancer, promoter, promoter barcode, enhancer barcode respectively.

ctgcgcgctcgctcgctcactgaggccgcccgggcaaagcccgggcggcctcagtgagcgagcgagcgcgcagagagggagtggccaactccatcactaggggttccttgtagttaatgattaacccgccatgctacttatctaccagggtaatggggatcctctagaactatagctagaattcgtttggtggcactggagcggccgcNNNNNNNNNNNNNNNNNNNNNNNNN
NNNNNNNNNNNNNNNNNNNNNNNNNNNNNNNNNNNNNNNNNNNNNNNNNNNNNNNNNNNNNNNNNNNNNNNNNNNNNNNNNNNNNNNNNNNNNNNNNNNNNNNNNNNNNNNNNNNNNNNNNNNNNNNNNNNNNNNNNNNNNNNNNNNNNNNNNNNNNNNNNN
NNNNNNNNNNNNNNNNNNNNNNNNNNNNNNNNNNNNNNNNNNNNNNNNNNNNNNNNNNNNNNNNNNNNNNNNNNNNNNNNNNNNNNNNNNNNNNNNNNNNNNNNNNNNNNNNNNNNNNNNNNNNNNNNNNNNNNNNNNNNNNNNNNNNNNNNNNNNNNNNNN
NNNNNNNNNNNNNNNNNNNNNNNNNNNNNNNNNNNNNNNatcgatNNNNNNNNNNNNNNNNNNNNNNNNNNNNNNNNNNNNNNNNNNNNNNNNNNNNNNNNNNNNNNNNNNNNNNNNNNNNNNNNNNNNNNNNNNNNNNNNNNNNNNNNNNNNNNNNNNNN
NNNNNNNNNNNNNNNNNNNNNNNNNNNNNNNNNNNNNNNNNNNNNNNNNNNNNNNNNNNNNNNNNNNNNNNNNNNNNNgccaccatggtgagcaagggcgaggaggataacatggccatcatcaaggagttcatgcgcttcaaggtgcacatggagggctccgtgaacggccacgagtttgagattgaaggcgaaggcgagggcagac
cctacgagggaacacagaccgccaagctgaaggtgaccaagggtggcccccctgcccttcgcctgggacatcctgtcccctcagttcatgtacggctccaaggcctacgtgaagcaccccgccgacatccccgactacttgaagctgtccttccccgagggcttcaagtgggagcgcgtgatgaacttcgaggacggcggcgtggtgaccgtgacccaggactcctccctgcaggacggcgagttca
tctacaaggtgaagctgcgcggcaccaacttcccctccgacggccccgtaatgcagaagaagacaatgggctgggaggcctcctccgagcggatgtaccccgaggacggcgccctgaagggcgagatcaagcagaggctgaagctgaaggacggcggccactacgacgctgaggtcaagaccacctacaaggccaagaagcccgtgcagctgcccggcgcctacaacgtcaacatcaagttg
gacatcacctcccacaacgaggactacaccatcgtggaacagtacgaacgcgccgagggtcgacactccaccggcggcatggacgagctgtacaagtagNNNNNNNNNNgcgatcgcNNNNNNNNNNggcgcgcctagccatctcgaggacggggtgaactacgcctgaggatccgatctttttccctctgccaaaaattatggggacatcatgaagccccttgagcatctgactt
ctggctaataaaggaaatttattttcattgcaatagtgtgttggaattttttgtgtctctcactcggaagcaattcgttgatctgaatttcgaccacccataatacccattaccctggtagataagtagcatggcgggttaatcattaactacaaggaacccctagtgatggagttggccactccctctctgcgcgctcgctcgctcactgaggccgggcgaccaaaggtcgcccgacgcccgggctttgcc
cgggcggcctcagtgagcgagcgagcgcgcag
